# Real-Time Monitoring of Apoptosis by Caspase-3-Like Protease Induced FRET Reduction Triggered by Amyloid Aggregation

**DOI:** 10.1155/2008/865850

**Published:** 2008-06-10

**Authors:** Johan F. Paulsson, Sebastian W. Schultz, Martin Köhler, Ingo Leibiger, Per-Olof Berggren, Gunilla T. Westermark

**Affiliations:** ^1^Department of Chemistry and The Skaggs Institute for Chemical Biology, The Scripps Research Institute, La Jolla, CA 92037, USA; ^2^Division of Cell Biology, Diabetes Research Centre, Department of Clinical and Experimental Medicine, Linköping University, 58185 Linköping, Sweden; ^3^The Rolf Luft Research Center for Diabetes and Endocrinology, Karolinska Institute, 17176 Stockholm, Sweden

## Abstract

Amyloid formation is cytotoxic and can activate the caspase cascade. Here, we monitor caspase-3-like activity as reduction of fluorescence resonance energy transfer (FRET) using the contstruct pFRET2-DEVD containing enhanced cyan fluorescent protin (EYFP) linked by the caspase-3 specific cleavage site residues DEVD. Beta-TC-6 cells were transfected, and the fluoorescence was measured at 440 nm excitation and 535 nm (EYFP) and 480 nm (ECFP) emission wavelength. Cells were incubated with recombinant pro lset Amyloid Polypeptide (*rec* prolAPP) or the processing metabolites of prolAPP; the N-terminal flanking peptide withIAPP (*rec*N+IAPP); IAPP with the C-terminal flanking peptied (*rec*IAPP+C) and lslet Amyloid Polypeptide (*rec*IAPP) . Peptides were added in solubilized from (50 *μ*M) or as performed amyloid-like fibrils, or as a combination of these. FRET was measured and incubation with a mixture of solubilized peptide and performed fibrils resulted in loss of FRET and apoptosis was determined to occure in cells incubated with *rec*proIAPP (49%), *rec*N+IAPP (46%), *rec*IAPP (72%) and *rec*IAPP+C (59%). These results show that proIAPP and the processing intermediates reside the same cell toxic capacity as IAPP, and they can all have a central role in the reduction of beta-cell number in type 2 diabetes.

## 1. INTRODUCTION

If proteins misfold or lose their native fold, they are usually assisted to refold by molecular chaperons or they can be removed from the system and be degraded [1]. However, under certain circumstances this system seems to fail and proteins escape degradation. Instead, they can aggregate into fibrils with a high degree of beta-pleated secondary structure, and if these fibrils are stained by Congo red and exert green birefringence when viewed in polarised light they are referred to as amyloid [2–5]. Amyloid deposition is associated with loss of organ function and cell death. Hitherto, at least 25 different proteins have been characterised from systemic or localised amyloid deposits in humans [4].

The amyloid formation process can be divided into two phases. The first is a nucleation forming process where monomers of the amyloid peptide form prefibrillar oligomeric species and this is referred to as the lag phase [6, 7]. The second phase is the extension phase where rapid elongation of amyloid fibrils occurs which will reach a plateau when most of the molecules are converted into the fibrillar form. A dramatic reduction of the lag phase will occur if mature amyloid fibrils of the same origin are added and this is called the seeding effect. There is growing evidence that the cytotoxic species of the amyloid forming process are early oligomers consisting of 15–40 monomers forming a ring shaped structure [8–10]. These prefibrillar assemblies are able to incorporate and form pores in cellular membranes causing cell leakage and influx of cations which triggers the apoptosis cascade. According to this, the amyloid fibril itself is a nontoxic end product of a cytotoxic aggregation process.

Amyloid deposits in the islets of Langerhans are a pathological characteristic of type 2 diabetes, and the amyloid aggregates consist of islet amyloid polypeptide (IAPP) [11, 12]. IAPP is a product of the endocrine beta-cell and as amyloid deposition occurs a large reduction of beta-cell mass has been observed [13–15]. IAPP has also been shown to form spherical structures with the capacity to incorporate into lipid bilayers [16–18]. In vitro studies of different cell lines show that amyloid formation of IAPP is cytotoxic and triggers the apoptotic pathway by activation of the caspase cascade [19–21].

IAPP is expressed as a precursor molecule (proIAPP) which is posttranslationally modified by the processing enzymes prohormone convertase (PC) 2 and 1/3 [22–25]. In the secretory granule, proIAPP is cleaved between dibasic residues at the N- and C-termini by PC2 and PC1/3, respectively. We and others have previously shown in cell lines that if aberrant processing of human proIAPP occurs, intracellular amyloid-like aggregates arise with cell death as a result [26, 27]. Also intracellular amyloid-like aggregates consisting of proIAPP have been described in both transgenic mice expressing human IAPP and in human beta-cells [28]. Cells with intracellular amyloid-like material are also positive for M30 cyto-death antibody which binds to an early neoepitope that becomes accessible during caspase activation.

Here, we describe a novel system for monitoring beta-cell apoptosis. The system uses two fluorophores linked together with a caspase 3-like cleavage site, and when cleavage occurs a reduction of fluorescence resonance energy transfer (FRET) can be measured as an indicator of apoptosis. Since measurements of the same cells can be done repeatedly, real-time monitoring of beta-cell apoptosis can be performed. Also, we used the established assay to compare the apoptotic properties of recombinant proIAPP (*rec*proIAPP) and proIAPP processing intermediates, N-terminal flanking peptide with IAPP (*rec*N+IAPP), IAPP with the C-terminal flanking peptide (*rec*IAPP+C), and recombinant IAPP (recIAPP).

## 2. EXPERIMENTAL PROCEDURES

### 2.1. Cell transfection

Beta-TC-6 (B-TC-6) cells obtained from American Type Culture Collection (Manassas, VA, USA) were cultured to 80% confluency in 10 cm diameter Petri dishes (Falcon: Labora, Stockholm, Sweden) in RPMI-1640 medium with 11 mM D-glucose containing 10% fetal bovine serum (FBS) (Sigma, Stockholm, Sweden), 100 IU/ml penicillin, 100 *μ*g/ml streptomycin, 50 *μ*M *β*-mercaptoethanol. One hour prior to transfection, medium was changed to RPMI-1640 medium without serum. A total volume of 175 *μ*L with 20 *μ*g pFRET2-DEVD, 20 *μ*g pcDNA3, 10 mM polyethyleneimine, and 5% sucrose was added to 9 mL of RPMI-1640 medium without serum. After 6 hours of incubation, FBS was added to a final concentration of 10%. After 24 hours, 0.4 mg/mL G-418 antibiotics was added to the medium for selection of stabile B-TC-6 clones.

### 2.2. Assay buffer

The following solutions were investigated for autofluorescence: (1) Krebs-Ringer with hepes and glucose (KRHG) (120 mM NaCl, 4.7 mM KCl, 2.5 mM CaCl_2_, 1.2 mM MgSO_4_, 0.5 mM KH_2_PO_4_, pH 7.4 with 2 mM D-glucose, 20 mM Hepes, and 200 nM adenosine). (2) Hank's balanced salt solution (HBSS) (5.4 mM KCl, 0.3 mM NaHPO_4_, 0.4 mM KH_2_PO_4_, 4.2 mM NaHCO_3_, 1.3 mM CaCl_2_, 0.5 mM MgCl_2_, 0.6 mM MgSO_4_, 137 mM NaCl, 5.6 mM D-glucose, pH 7.4). (3) RPMI-1640 medium without FBS (Sigma). (4) RPMI-1640 medium with 10% FBS. (5) Dulbeccoçs medium without phenol red (Invitrogen, Carlsbad, Calif, USA). (6) Schneiderçs Drosophila medium without phenol red (Invitrogen). (7) H_2_0.

### 2.3. Real-time monitoring of apoptosis

pFRET2-DEVD vector is driven by the insulin promoter and can only be expressed in insulin producing cells. When expressed, a product consisting of enhanced cyan fluorescent protein (ECFP) and enhanced yellow fluorescent protein (EYFP) linked by the amino acid residues DEVD will be dispersed throughout cytoplasm. The sequence DEVD is a specific substrate for caspase-3-like proteases and when ECFP and EYFP are connected by the DEVD residues FRET will occur. If the transfected B-TC-6 cells undergo apoptosis, the DEVD is cleaved and the two fluorophores will be separated and a loss of FRET can be monitored as a decrease of the 535 nm/480 nm fluorescence ratio. The pFRET2-DEVD vector has previously been characterised by Köhler et al. [29]. B-TC-6 cells with stable expression pFRET2-DEVD were cultured for 48 hours in black 96 well plates (Labsystems) to 80% confluency and washed once in KRHG-buffer prior to experimental procedures. Sample volume was set to 100 *μ*L and both synthetic and recombinant peptides were kept as stock solution of 5 mM in dimethyl sulfoxide (DMSO) and diluted to a final concentration of 50 *μ*M with a final DMSO concentration of 1%. Synthetic human and rat IAPP were analysed in solubilised form at final concentrations of 25 and 50 *μ*M. Negative control was 1% DMSO in KRHG-buffer and when seeded assays were performed a final concentration of 30 nM synthetic IAPP fibrils was included in the negative control. Positive control for apoptosis contained 2 *μ*M staurosporine and 1% DMSO in KRHG-buffer. Negative and positive controls were included in each individual assay. Mature recombinant amyloid-like fibrils were washed in H_2_O and centrifuged at 16.000 g for 15 minutes and resuspended in KRHG-buffer, sonicated and diluted to an estimated concentration of 50 *μ*M.

Loss of FRET was measured in a Wallac 1420 multilabel counter (Perkin Elmer, Turku, Finland) with WorkOut software version 1.5 (Perkin Elmer). Excitation was set to 440 nm, and emitted fluorescence was measured at 535 nm (EYFP) and 480 nm (ECFP). Data are presented as mean ratio ±SEM of 535 nm/480 nm ratio. Each point represents at least five individual measurements.

### 2.4. Confocal microscopy

B-TC-6 cells with stabile expression of pFRET2-DEVD and untransfected B-TC-6 cells were cultured on 19 mm cover slips and rinsed in PBS (137 mM NaCl, 2.7 mM KCl, 4.3 mM Na_2_HPO_4,_ and 1.4 mM, KH_2_PO_4_) before fixation in 2% paraformaldehyde in PBS for 30 minutes. For visualisation, cells were incubated with the nuclear stain TO-PRO-3 (Molecular probes, Eugene, Ore, USA) diluted 1 : 1000 in PBS for 15 minutes and then mounted with 50/50 glycerol/PBS. Cells were studied in a Nikon eclipse E600 microscope with a Nikon C1 confocal unit with argon 488 nm and HeNe 633 nm lasers (Nikon Kawasaki, Japan). Digital pictures were taken with an EZ-C1 digital camera and software version 1.0 for Nikon confocal microscopy.

### 2.5. Production of recombinant peptides

Human preproIAPP cloned into the pBluescript II vector was used as template for generation of PCR amplified DNA fragments corresponding to human proIAPP, N-terminal flanking peptide+IAPP, IAPP and IAPP+C-terminal flanking peptide. Forward and reverse primers used for this purpose are described in Table 1. The amplified IAPP fragments were blunt end ligated in expression vector pGEX 2TK (GE healthcare, Uppsala, Sweden). Constructs were confirmed by sequencing to be in correct reading frame and without mutations. The pGEX 2TK vector has glutathione S-transferase (GST) in front of the multiple cloning site and the peptide will be expressed as a GST fusion protein. Y1090 bacteria were transformed and cultured in Luria broth at +37°C until OD A_600_ reached 0.8, and protein synthesis was induced with 3 mM isopropyl *β*-D-1 thiogalactopyranoside (IPTG) (Fermentas, St Lenon Rot, Germany) for 3 hours at +25°C. Bacteria were spun down and resuspended in TEDG buffer (50 mM TRIS-HCl pH 7.4, 1.5 mM EDTA, 10% glycerol, 400 mM NaCl) and sonicated three times for 20 seconds. The bacteria lysate was centrifuged at 100.000 g in a SW41 Ti rotor for 30 minutes at +4°C, and the supernatant was transferred to Sepharose-4B beads (GE healthcare) and incubated 2 hours end over end at +4°C. Sepharose beads were spun down at 3000 g for one minute, and the lysate was decanted and the beads were washed three times in NET-N buffer (50 mM TRIS-HCl pH 7.4, 150 mM NaCl, 5 mM EDTA and 0.5% NONIDET-NP 40 (USB, Cleveland, Ohio, USA) followed by three times wash in PBS. The GST-tag was cleaved off with thrombin protease (GE healthcare) 20 U/mg expected peptide in PBS end over end overnight. Removal of the 27 kDa GST-tag resulted in rapid amyloid formation and aggregates were collected. The amyloid-like fibrils were washed in ddH_2_O and solved in 50%/50% 1,1,1,3,3,3-hexafluoro-2-propanol (HFIP)/trifluoroacetic acid (TFA) for 24 hours. Solubilised peptides were centrifuged at 16.000 g for 15 minutes, and the supernatant was recovered. For assays with IAPP recombinant peptides in complete monomeric form, the supernatant was filtered trough a Millex-FG syringe filter 0.2 *μ*m for hydrophobic solvents. Samples were dried in vacuum and redisolved in 100% DMSO. Peptide concentrations for *rec*proIAPP and *rec*N+IAPP were determined using A_280_ extinction coefficient 1615 M^−1^ cm^−1^and for *rec*IAPP and *rec*IAPP+C 3105 M^−1^ cm^−1^ in a nanodrop ND-1000 spectrophotometer (NanoDrop Technologies, Wilmington, Del, USA). Theoretical molecular masses were as follow: *rec*proIAPP 8358 Da, *rec*N+IAPP 6224 Da, *rec*IAPP 4918 Da, and *rec*IAPP+C 7053 Da. Synthetic human IAPP used in the study was synthesized by KEX Laboratories, Yale University (New Haven, Conn, USA), and synthetic rat IAPP was purchased from Bachem (Heidelberg, Germany).

### 2.6. Tricine-sodium dodecyl sulfate-polyacrylamide gel electrophoresis

Tricine-SDS-PAGE was performed as described by Schägger and von Jagow [30]. Samples were dissolved in sample buffer containing 0.1 M Tris-HCl at pH 6.8, 30% (wt/vol) glycerol, 8% (wt/vol) SDS, 0.2 M dithiothreitol (DTT), and 0.02% Coomassie blue G-250 and boiled for 5 minutes prior to loading onto the gel. The separation condition was 30 mV for 20 hours.

### 2.7. Silver staining and trypsin digestion

The tricine-SDS gel was sensitised in 1.4 mM Na_2_S_2_O_4_ and incubated in 0.2% AgNO_3_ solution containing 7.5 *μ*L formaldehyde (37%)/100 mL water for 25 minutes and developed in 6% Na_2_CO_3_, 25 *μ*M Na_2_S_2_O_3_, and 50 *μ*L formaldehyde (37%)/100 mL water.

Silver stained bands corresponding to the expected masses of the recombinant IAPP peptides were excised from the gel and dried. Each sample was incubated with 60 *μ*L reducing agent consisting of 10 mM dithiothreitol (DTT) in 25 mM NH_4_HC_3_ for one hour at +56°C, after which the peptides were alkylated in 70 *μ*L 55 mM iodoacetamide (IAA) at room temperature in dark for 45 minutes. Rehydrated gel pieces were digested in 100 *μ*L trypsin solution containing 1.5 *μ*g/mL trypsin (Promega, Madison, Wis, USA) and 25 mM NH_4_HCO_3_ and incubated 24 hours at +37°C.

### 2.8. Electro spray ionisation tandem mass spectrometry

Full scan mass spectra for peptide mass fingerprinting and TOF product scans for sequencing of amino acids were acquired on a hybrid mass spectrometer (API QSTAR Pulsar, Applied Biosystems, Foster city, Calif) equipped with a nanoelectrospray ion source (MDS Protana, Odense, Denmark).

### 2.9. Amyloid staining

Samples were dried onto glass slides and incubated for 20 minutes in A solution (NaCl saturated 80% ethanol with 0.01% NaOH) and were directly transferred to B solution (solution A saturated with Congo red). Samples were rinsed in 100% ethanol and xylen and mounted. Amyloid specific green birefringence was studied in an Olympus X51 microscope (Olympus, Tokyo, Japan) with two polarising filters connected to an Olympus DP 50 digital camera run by Studio Lite v 1.0.1 software.

### 2.10. Electron microscopy

Droplets of sample were placed on formvar coated copper grids and were negative contrasted with 2% uranyl acetate in 50% ethanol. Samples were studied in a Jeol 1230 electron microscope at 100 kV (Jeol, Akishima, Tokyo, Japan). Digital electron micrographs were taken with a Gatan multiscan camera model 791 with corresponding software v3.6.4 (Gatan Inc, Pleasanton, Calif, USA).

### 2.11. Thioflavin Tassay

Kinetic studies of amyloid formation were performed in Sigmacote (Sigma-Aldrich, St. Louis, Mo, USA) treated black 96 well plates (Labsystems) in a sample volume of 100 *μ*L. Synthetic IAPP was diluted from a DMSO stock solution (5 mM) to a final concentration of 50 *μ*M in thioflavin T (ThT) in assay buffer (50 mM glycine, 25 mM sodium phosphate buffer, pH 7.0, and 10 *μ*M ThT). Mature IAPP fibrils (30 nM) were included to decrease the amyloid lag phase. Samples consisting of ThT assay buffer with 1% DMSO and ThT assay buffer with 30 nM IAPP fibrils and 1% DMSO were included in the assay. Fluorescence was measured every thirty minutes at an excitation wavelength of 442 nm and an emission wavelength of 486 nm. Each sample was measured in 12 individual wells, and data presented are mean values ± SEM.

### 2.12. Statistical analysis

Statistics were performed in GraphPad InStat version 3.06 (GraphPad Software Inc., San Diego, Calif, USA). Unpaired *t*-test was used when two groups were compared and one-way analysis of variance (ANOVA) with Dunnett multiple comparisons test when several groups were analysed. A *P*-value less than 0.05 was considered significant.

## 3. RESULTS

### 3.1. Evaluation of the method

**Figure 1:**
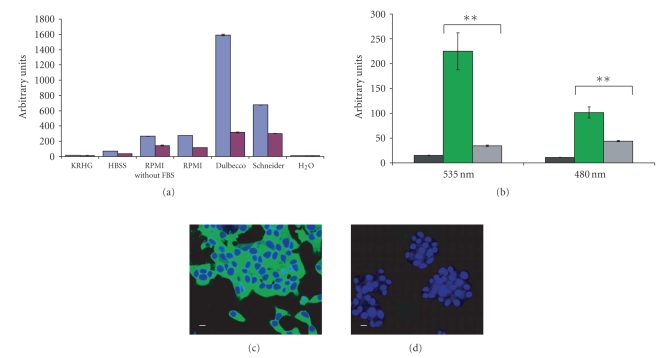
Determination of assay buffer and investigation of pFRET2-DEVD expressing B-TC-6 cells. (a) Solutions (KRHG, HBSS, RPMI without FBS, RPMI, Dulbeccoçs modified medium, Schneiderçs drosophila medium, and H_2_O) were analysed for autofluorescence at wavelength 535 nm (blue columns) and 480 nm (red columns). Data presented are mean values ±SEM (*n* = 8). (b) Fluorescence at 535 nm and 480 nm was measured for KRHG buffer (black columns), B-TC-6 cells expressing pFRET2-DEVD (green columns), and untransfected B-TC-6 cells (grey columns). Data presented are mean values ±SEM (*n* = 8). Difference in fluorescence signal between B-TC-6 cells expressing pFRET2-DEVD and untransfected B-TC-6 was considered to be significant (*P* < .0005) in a two-tailed unpaired *t*-test. (c) Confocal image of pFRET2-DEVD expressing B-TC-6 cells and (d) untransfected B-TC-6 cells. Argon 488 nm and HeNe 633 nm lasers were used at the same energy levels in the two images. Green fluorescence indicates expression of pFRET2-DEVD and blue fluorescence nuclear staining TO-PRO-3. Scale bars represent 10 *μ*m.

Autofluorescence from different solutions was analysed at emission wavelengths 535 nm and 480 nm (see Figure 1(a)). The KRHG buffer had the lowest fluorescence of the investigated solutions and was therefore used as assay buffer throughout the study.

To determine if the fluorescent signal from the pFRET2-DEVD transfected B-TC-6 cells was sufficient for detection in the plate reader the signal was compared to the signal from untransfected B-TC-6 cells. B-TC-6 cells expressing pFRET2-DEVD had a 6.5-fold higher fluorescence signal at 535 nm and a 2.3-fold higher signal at 480 nm when compared to the untransfected B-TC-6 cells (see Figure 1(b)). Fluorescence form transfected B-TC-6 cells differed significantly at both wavelengths compared to untransfected B-TC-6 cells in an unpaired *t*-test (*P* < .0005). The 535 nm/480 nm ratio for living pFRET2-DEVD transfected B-TC-6 cells was 2.2. Three hours incubation with the apoptosis inducer staurosporine (2 *μ*M) decreased the FRET ratio to 1.2. Synthetic human and rat IAPP peptides in monomeric form, at concentrations 25 and 50 *μ*M, were incubated with pFRET2-DEVD B-TC-6 cells. Only negligible reduction in FRET signal was detected after 12 hour incubation, and cell survival was calculated to 96% and 98% after incubation with rat IAPP and human IAPP, respectively. There was no difference between cells incubated with 25 or 50 *μ*M (data not shown). In the confocal microscope, the pFRET2-DEVD B-TC-6 cells revealed a strong cytoplasmic fluorescence at excitation wavelength 488 nm (see Figure 1(c)). This was not present in untransfected cells (see Figure 1(d)).

### 3.2. Amyloid formation and loss of FRET

**Figure 2:**
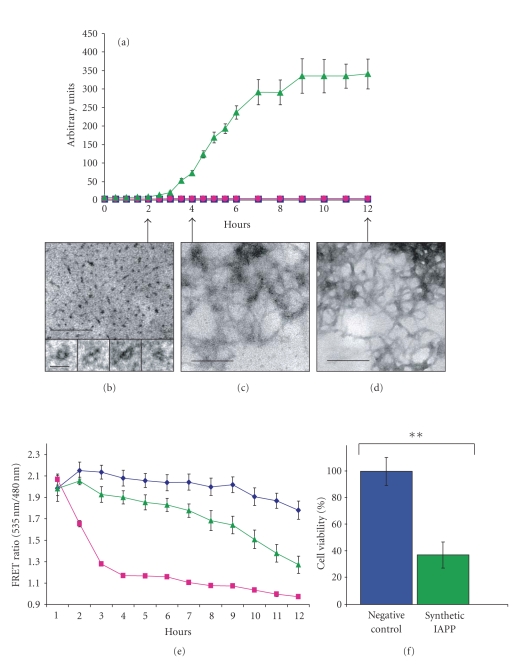
Parallel performed ThT assay, electron microscopy, and FRET assay of synthetic IAPP peptide. (a) ThT assay of synthetic IAPP peptide (50 *μ*M) seeded with preformed IAPP fibrils (30 nM) (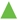), ThT assay buffer seeded with preformed IAPP fibrils (30 nm) (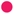), and ThT assay buffer (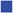). Data presented are mean values ±SEM (*n* = 12). Synthetic IAPP peptide (50 *μ*M) seeded with preformed IAPP fibrils (30 nM) incubated for (b) 2 hours of 50 *μ*M, (c) for 4 hours, (d) and for 12 hours; all samples were negative contrasted. In (b), the higher magnification shows small prefibrillar ring shaped structures. In (c), thin thread-like protofibrils are visible and in (d) more mature amyloid-like fibrils are apparent. Large-scale bars represent 100 nm, and the smaller-scale bar in (b) represents 20 nm. (e) FRET ratio of 535 nm/480 nm measurements of KRHG buffer with seeds of preformed IAPP fibrils (30 nM) (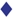), synthetic IAPP peptide (50 *μ*M) seeded with preformed IAPP fibrils (30 nM) (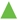), and staurosporine (2 *μ*M) (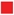). Measurements were performed during a 12-hour period with individual measurements each hour. Data presented are mean values ±SEM (*n* = 9). (f) FRET ratio after 12 hours of measurements. Staurosporine sample was set as zero and was subtracted from assay measurements, and the negative control was set as 100% viable cells (blue column). The FRET ratio of the B-TC-6 cells incubated in synthetic IAPP peptide (50 *μ*M) seeded with preformed IAPP fibrils (30 nM) was 37% of the negative control corresponding to a population of 65% apoptotic cells (green column). Data presented are mean values ±SEM (*n* = 9). The difference between the negative control and IAPP was considered significant in a two-tailed unpaired *t*-test (*P* < .0006).

FRET reduction as a result of amyloid formation was initially investigated with synthetic IAPP peptides. The amyloid formation from 50 *μ*M monomeric IAPP seeded with 30 nM IAPP fibrils was monitored in the ThT assay and with the FRET assay in parallel (see Figure 2). In the ThT assay, amyloid specific fluorescence of seeded IAPP was detected after a 3-hour lag phase and reached a plateau after 9 hours (see Figure 2(a)). No increase in basal fluorescence was detected for the sample with ThT buffer alone or ThT buffer with low concentration of premade IAPP seeds. To further study the amyloid aggregation process, aliquots from the seeded 50 *μ*M IAPP solution were collected hourly and prepared for electron microscopy. After 2 hours, small ring shaped structures with an outer diameter of approximately 15 nm could be detected at high magnification (see Figure 2(b)). After 4 hours of incubation, very thin thread-like fibrils were detected (see Figure 2(c)) and after 12 hours of incubation fibrils with a more mature fibrillar appearance were visible (see Figure 2(d)).

In the FRET measurements, pFRET2-DEVD expressing B-TC-6 cells incubated with staurosporine used as positive control showed an instant reduction of the 535 nm/480 nm ratio. After 4 hours, the ratio was reduced to 1.2 (see Figure 2(e)). The FRET ratio for pFRET2-DEVD expressing B-TC-6 cells incubated with KRHG buffer and 30 nM IAPP seeds used as negative control were relative stable at a ratio of 2.0 through out the assay. pFRET2-DEVD expressing B-TC-6 cells incubated in 50 *μ*M IAPP peptide with 30 nM IAPP seeds showed a reduced FRET signal compared to the negative control (see Figure 2(e)). The measurements were done hourly for 12 hours, and the calculation was performed as follows. The FRET ratio 535 nm/480 nm, determined after 12 hours of incubation with the apoptosis-inducer staurosporine, was set as baseline and corresponds to 0% survival cells. All cells were considered viable after incubation with preformed fibrils, and the value for the FRET ratio 535 nm/480 nm was set as 100% after subtraction of the baseline value.

The FRET ratio of the B-TC-6 cells incubated in amyloid forming IAPP peptide corresponds to a population of 65% apoptotic cells (see Figure 2(f)). B-TC-6 cells incubated in amyloid forming IAPP differed significantly from the negative control in a two-tailed unpaired *t*-test (*P* < .006).

### 3.3. Characterisation of recombinant peptides

**Figure 3:**
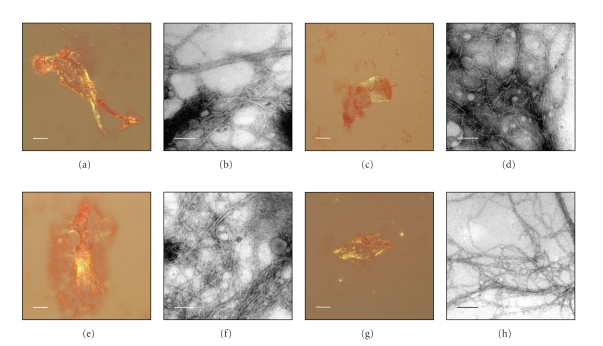
Congo red staining and electron micrographs of amyloid-like fibrils from *rec*proIAPP, *rec*N+IAPP, *rec*IAPP, and *rec*IAPP+C. Congo red staining viewed in polarised light showing amyloid specific green birefringence of (a) *rec*proIAPP, (c) *rec*N+IAPP, (e) *rec*IAPP, and (g) *rec*IAPP+C. Scale bars represent 20 *μ*m. Electron micrographs of negative contrasted amyloid-like fibrils of (b) *rec*proIAPP, (d) *rec*N+IAPP, (f) *rec*IAPP, and (h) *rec*IAPP+C. Scale bars represent 100 nm.

The recombinant peptides *rec*proIAPP, *rec*N+IAPP, *rec*IAPP, and *rec*IAPP+C were expressed as fusion proteins and when the GST-tag was enzymatically removed, all four peptides formed aggregates during the 12 hours time period. The aggregates from the different recombinant peptides stained with Congo red and revealed green birefringence (see Figures 3(a)–3(d)). Negative contrasted aggregates showed typical amyloid-like fibrils with a diameter of 10 nm and of variable length (see Figures 3(e)–3(h)).

**Figure 4:**
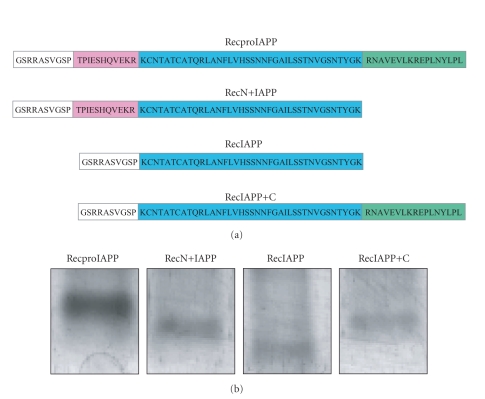
Characterisation of *rec*proIAPP, *rec*N+IAPP, *rec*IAPP, and *rec*IAPP+C. (a) A cartoon showing the amino acid sequences of *rec*proIAPP, *rec*N+IAPP, *rec*IAPP, and *rec*IAPP+C peptides. N-terminally, a fragment consisting of 10 residues originating from the GST-tag is present (white box). (b) Silver stained tricine SDS-PAGE of *rec*proIAPP, *rec*N+IAPP, *rec*IAPP, and *rec*IAPP+C. Bands were removed from the tricine gel, trypsinised and analysed by electrospray ionisation tandem mass spectrometry.

The expected amino acid sequences of *rec*proIAPP, *rec*N+IAPP, *rec*IAPP, and *rec*IAPP+C was used to determine the expected molecular masses (see Figure 4(a)).The produced peptides were run on Tricine SDS-PAGE and bands corresponding to the molecular masses of each individual peptide were cut out, trypsinised and analysed by electrospray ionisation tandem mass spectrometry (see Figure 4(b)). The expected tryptic fragments from each peptide were identified in full scan spectrum (data not shown), and the sequence of each fragment was confirmed by collision-induced fragmentation mass spectrometry. All four IAPP peptides had a 10-amino acid residue sequence consisting of residues GSRRASVGSP N-terminally which is a rest from the GST cleavage site. These extra residues did not interfere with the capability of the recombinant peptides to form amyloid-like fibrils as shown in Figure 3.

### 3.4. Apoptotic effect of recombinant peptides

**Figure 5:**
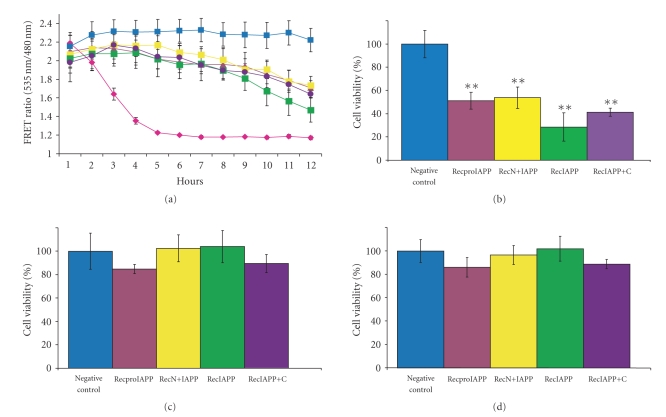
*FRET assays of* *rec*proIAPP, *rec*N+IAPP, *rec*IAPP, and *rec*IAPP+C. (a) B-TC-6 cells expressing pFRET2-DEVD were incubated with solubilised recombinant peptides *rec*proIAPP (50 *μ*M) (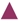), *rec*N+IAPP (50 *μ*M) (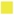), *rec*IAPP (50 *μ*M) (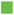), and *rec*IAPP+C (50 *μ*M) (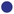), all supplemented with preformed IAPP fibrils at a concentration of 30 nM or incubated with preformed IAPP fibrils (30 nM) alone (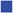), or staurosporine (2 *μ*M) (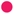). The FRET ratio at 535/480 nm was determined hourly over 12 hours. Data presented are mean value ±SEM (*n* = 5). (b) Cell viability after incubation for 12 hours with solubilised recombinant peptides supplemented with preformed IAPP fibrils (30 nM). Data are presented as mean value ±SEM (*n* = 5). Relative to the control, cell viability was determined to 51% for *rec*proIAPP (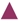), 54% for *rec*N+IAPP (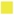), 28% for *rec*IAPP (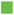), and 41% for *rec*IAPP+C (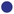). Compared to the control, a significant difference in the FRET ratio was observed for all four recombinant peptides in an ANOVA test (*P* < .0005). (c) FRET ratio of pFRET2-DEVD expressing B-TC-6 cells after 12 hours of incubation with recombinant amyloid-like fibrils corresponding to 50 *μ*M. *Rec*proIAPP (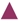), *rec*N+IAPP (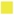), *rec*IAPP (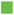), and *rec*IAPP+C (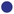) and control incubation with buffer alone (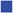). Relative to the control, cell viability after incubation with *rec*proIAPP was 85%, with *rec*N+IAPP 102%, with *rec*IAPP 104% and with *rec*IAPP+C 89%. No statistically difference between the negative control and the four recombinant peptides was observed in an ANOVA test (*P* < .5). (d) Incubation of pFRET2-DEVD expressing B-TC-6 cells with recombinant peptides in solubilised form (50 *μ*M). *Rec*proIAPP (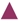), *rec*N+IAPP (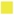), *rec*IAPP (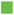), and *rec*IAPP+C (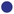) and control incubation with buffer alone (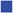). Relative to the control, cell viability after 12 hours incubation with *rec*proIAPP was 86%, *rec*N+IAPP 97%, *rec*IAPP 102%, and *rec*IAPP+C 89%. The four recombinant peptides did not differ significantly from the negative control in an ANOVA test (*P* > .06).

pFRET2-DEVD expressing B-TC-6 cells were incubated with 50 *μ*M of *rec*proIAPP, *rec*N+IAPP, *rec*IAPP, and *rec*IAPP+C together with 30 nM IAPP seeds in KRHG buffer. A reduction of the FRET ratio was detected during the time laps of the assay (see Figure 5(a)). After 12 hours of incubation, all four recombinant peptides had generated a large reduction of the FRET signal. The FRET ratio of the positive control was set as zero and subtracted from all other measurements and the negative control was set as 100% viable cells. The FRET ratios for *rec*proIAPP, *rec*N+IAPP, *rec*IAPP, and *rec*IAPP+C were 51%, 54%, 28%, and 41%, respectively, when compared to the negative control. This corresponds to a population of 49%, 46%, 72%, and 59% apoptotic cells (see Figure 5(b)). All four groups differed significantly from the negative control in an ANOVA test (*P* < .0005). No significant difference within the four recombinant peptide groups was detected in an ANOVA test (*P* > .2).

pFRET2-DEVD expressing B-TC-6 cells was also incubated with mature amyloid-like fibrils from *rec*proIAPP, *rec*N+IAPP, *rec*IAPP, and *rec*IAPP+C. No difference in FRET ratio was observed over time when compared to the negative control (data not shown). After 12 hours of incubation, FRET ratios of *rec*proIAPP, *rec*N+IAPP, *rec*IAPP, and *rec*IAPP+C were 85%, 102%, 104%, and 89%, respectively, when the positive control was subtracted and the negative control was set as 100% viable cells (see Figure 5(c)). No significant differences between the negative control and the recombinant fibrils and no difference between the four different peptides (*P* > .5) were detected.

FRET measurements were also performed on pFRET2-DEVD expressing B-TC-6 cells incubated with 50 *μ*M solubilised and filtered recombinant peptides. When compared to the negative control, no loss of FRET was detected during 12 hours of measurements (data not shown). After 12 hours of incubation and subtraction of the positive control, FRET ratios for *rec*proIAPP, *rec*N+IAPP, *rec*IAPP, and *rec*IAPP+C were 86%, 97%, 102%, and 89%, respectively, compared to the negative control (see Figure 5(d)). No significant difference between the negative control and the monomeric peptides and no differences between the four different recombinant peptides (*P* > .06) were detected.

## 4. DISCUSSION

The pFRET2-DEVD vector has earlier been demonstrated by Köhler et al. to be an excellent reporter for apoptosis induced by caspase-3-like proteases in the insulin producing beta-cell lines RINm5F and MIN6 [29]. By measuring loss of FRET on individual transiently transfected cells, the course of apoptosis was monitored in real-time. Here, we used the pFRET2-DEVD vector to establish a novel assay with stable transfected B-TC-6 cells that allows accurate real-time studies of apoptosis in 96 well plates. The assay was used for studies of beta-cell apoptosis during amyloid formation, and the toxicity of *rec*proIAPP and the processing metabolites *rec*N+IAPP, *rec*IAPP, and *rec*IAPP+C is evaluated.

Initially, it was essential to find an assay buffer with very low autofluorescence for measurement of FRET signal from cells cultured in a black 96 well plate. The KRHG-buffer revealed the lowest background and was selected for the assay, but with the disadvantage of being a rather poor medium. After 12 hours of FRET measurement, the negative control started to show some reduction of FRET and therefore measurements beyond this time point are not presented throughout this work. The fluorescent signal detected from the pFRET2-DEVD transfected B-TC-6 cells was adequate for plate reader measurements and since it is the ratio between 535 and 480 nm, that is, given differences in cell density can be ignored. Synthetic IAPP peptide was initially used for the setup of the method.

One step in the evaluation procedure of the FRET method was to determine the amyloid fibril formation progress under the used peptide concentrations and correlate it to the induction of apoptosis. For this, a ThT assay was run in parallel with identical IAPP concentrations. When monomeric IAPP was seeded with preformed IAPP amyloid-like fibrils, an increase in ThT-fluorescence signal appeared after a three hours lag phase and reached maximum after nine hours of incubation. Fibril formation could only be detected in seeded IAPP samples, and not in samples with seed alone. In samples collected for morphological evaluation, an amorphous material was present after two hours and at higher resolution ring-like structures with an outer diameter of 15 nm could be seen. After four hours of incubation, thin slender fibrils were present. Since no fibrilar material was present at the earlier time points we conclude that these thin fibrils were newly formed and did not represent the low amount of sonicated preformed fibrils added as seed. In samples taken after 12 hours, fibrils with a more distinct fibrilar appearance had emerged. The decrease in FRET ratio after incubation with 2 *μ*M staurosporine starts under the first hour and continues over a five-hour period. Staurosporine is a potent inducer of apoptosis, a transient biological process that persists for 3-4 hours. In this positive control, all cells undergo apoptosis. In transfected cells incubated with monomeric IAPP and preformed fibrils, apoptosis was initiated after approximately seven hours and this is in line with the expected time frame if oligomers are responsible for the induction of apoptosis.

The B-TC-6 cell line is of mouse origin, and it has previously been suggested that mouse IAPP has an antiamyloidogenic effect on human IAPP at least intracellularly [31]. In vitro studies show that insulin also has an inhibitory property on amyloid formation of human IAPP [32, 33]. Prior to the experimental procedure, the B-TC-6 cells were washed in KRHG buffer which has a low glucose concentration (2 mM). Therefore, exocytosis of mouse IAPP and insulin was considered to be very low during the assay and not to influence the amyloidogenicity of human IAPP.

The predominant hypothesis of amyloid cytotoxicity is that the prefibrillar assemblies are able to incorporate into cellular membranes causing cation influx [10, 34, 35]. Oligomeric species from different peptides would there share a common structure and act cytotoxic by the same mechanisms. A common pathway for apoptosis triggered by amyloid pore formation has not yet been proposed. Apoptosis is the major cause of beta-cell reduction in type 2 diabetic patients and has been shown to occur during formation of islet amyloid in a transgenic mouse model [15, 36]. Two apoptosis studies on beta-cell lines have been performed where activation of the caspase cascade in parallel to IAPP amyloid formation has been observed [19, 20]. Both authors have used RINm5F cells and Zhang el al. have also used the human insulinoma cell line CM. Both groups reported the activation of the common downstream protease caspase 3 with upstream activated JNK pathway (c-jun NH2-terminal kinase/stress-activated protein kinase). Zhang et al. also reported activated caspase 8 and caspase 1 proteases upstream of caspase 3. The exact pathways of IAPP triggered apoptosis are not clear but caspase 3 acts as a common downstream effector. Zhang et al. state that caspase 3 activation was detected after 8 hours of incubation with preseeded synthetic IAPP and reached a maximum after 16 hours [20]. No measurements between these time points were performed. Our observation of the reduced FRET signal goes well with these data, and upstream activation must occur before we detect FRET reduction by caspase 3 activation.

All four recombinant peptides, *rec*proIAPP, *rec*N+IAPP, *rec*IAPP, and *rec*IAPP+C possess amyloidogenic properties and form fibrillar aggregates. The extra amino acid residues N-terminally of the recombinant peptides do not interfere with the amyloidogenic propensity since all four peptides form amyloid-like fibrils. The apoptotic effects of the recombinant peptides were investigated using the developed FRET assay. The recombinant peptides were studied in solubilised seeded, fibrillar, and in solubilised nonseeded form. A loss of FRET was detected for all four seeded solubilised recombinant peptides. At the end of the assay, an apoptotic rate ranging form 46–72% was observed for the four different seeded recombinant peptides though no statistically significant difference between the groups was distinguished. Important, the degree of apoptosis did not differ between cells incubated with synthetic IAPP (65%) or *rec*IAPP (72%) (*P* > .5) pointing out that the addition of the 10 residues at the N-terminus of the recombinant IAPP did not interfere with the cytotoxicity of the peptide.

B-TC-6 cells expressing pFRET2-DEVD were incubated with mature amyloid fibrils from *rec*proIAPP, *rec*N+IAPP, *rec*IAPP, and *rec*IAPP+C and no apoptosis was detected. Since the amyloid-like fibrils were extensively washed with water, a pure fibril fraction without any oligomeric species would be expected. Solubilised nonseeded recombinant peptides did not elicit a reduction of the FRET signal, most likely due to a long lag phase. From this we conclude that the cytotoxic species of synthetic and recombinant amyloidogenic IAPP peptides are early oligomers or protofibrils. This is in consensus with earlier studies [16, 17, 37].

A new and very interesting finding is that *rec*proIAPP and the processing intermediates *rec*N+IAPP and *rec*IAPP+C trigger beta-cell apoptosis. Earlier we and others have shown that islet amyloid deposition may start intracellularly and that aberrant processing of proIAPP can be an initiating event [26, 27]. Intracellular deposits containing proIAPP have been found both in transgenic mice expressing human IAPP and in human beta-cells transplanted under the renal capsule of mice fed a high fat diet [28]. In the latter study, we also found intra-granular amyloid-like fibrils consisting of proIAPP. It has previously been shown that amyloid formation of IAPP was strongly enhanced by phospholipid bilayers and that amyloid elongation occurred from the surface of the lipid bilayer [38]. Hypothetically, the membrane of the secretory granule or endoplasmatic reticulum may be the location of initial amyloid formation and where oligomeric pore structures can incorporate. By exocytosis, the granule membrane will fuse with the outer cell membrane and a cation influx and activation of apoptotic pathways might occur. Here, we demonstrate that *rec*proIAPP and the processing intermediates *rec*N+IAPP and *rec*IAPP+C can induce caspase 3 activation and trigger beta-cell apoptosis. This is achieved by prefibrillar toxic species since fibrillar and monomeric forms of the recombinant peptides did not trigger caspase 3-like activity. This further strengthens our hypothesis that aberrant processing of proIAPP plays a key role in early islet amyloidogenesis.

Apoptosis is a transient event, and the established assay allows analysis over time of the same cell population. It is a system with high reproducibility, and it is cost effective and easy to handle. Apoptosis assays where Ac-DEVD-AMC is used as fluorogenic substrate is often labour intense and the commonly used MTT assay, where only living cells are measured, is not a true assay for apoptosis. The Vybrant apoptosis detection kit uses three different fluorescent nuclear stains with the propensity to penetrate cell membrane according to an apoptotic or nonapoptotic state. This method is performed on living cells and must be analysed microscopically. It is work intense and dependent on the interpretation of the analyst. Also TUNEL and DNA fragmentation methods are labour intense and similar to all the methods described above, only measurement at one time point is possible. Factors such as equal amounts of cells in each well or assay and rate of cell division are very important to consider when to deal with the described methods. The weakness of our FRET assay is the poor medium in which the assay is performed. Further investigations of possible media to prolong the survival of the cells and thereby allow measurements over a more extended period of time would be valuable. Future prospects for this method are to study apoptosis in presence of antiamyloidogenic or amyloid enhancing factors depending on strategy to reduce toxic species in the amyloid forming process.

## Figures and Tables

**Table 1:** Forward and reverse primers for amplification of IAPP fragments.

proIAPP forward	5′-GAT GAC ACC CAT TGA AAG TCA TCA GG-3′
proIAPP reverse	5′-CTA CTA AAG GGG CAA GTA ATT CAG TGG-3′
N-terminal+IAPP forward	5′-GAT GAC ACC CAT TGA AAG TCA TCA GG-3′
N-terminal+IAPP reverse	5′-CTA CTA GCC ATA TGT ATT GGA TCC CAC G-3′
IAPP forward	5′-GAT GAA ATG CAA CAC TGC CAC ATG-3′
IAPP reverse	5′-CTA CTA GCC ATA TGT ATT GGA TCC CAC G-3′
IAPP+C-terminal forward	5′-GAT GAA ATG CAA CAC TGC CAC ATG-3′
IAPP+C-terminal reverse	5′-CTA CTA AAG GGG CAA GTA ATT CAG TGG-3′

## ABBREVIATIONS

DMSO: Dimethyl sulfoxide
ECFP: Enhanced cyan fluorescent protein
EYFP: Enhanced yellow fluorescent protein
FBS: Fetal bovine serum
FRET: Fluorescence resonance energy transfer
GST: Glutathione S-transferase
HBSS: Hank's balanced salt solution
IAPP: Islet amyloid polypeptide
IPTG: Isopropyl *β*-D-1 thiogalactopyranoside
KRHG: Krebs-Ringer salt solution with hepes and glucose
PC: Prohormone convertase
*rec*IAPP: Recombinant islet amyloid polypeptide
*rec*IAPP+C: Recombinant islet amyloid polypeptide with C-terminal flanking peptide
*rec*N+IAPP: Recombinant islet amyloid polypeptide with N-terminal flanking peptide
*rec*proIAPP: Recombinant pro islet amyloid polypeptide
ThT: Thioflavin T

## References

[1] Bukau B, Weissman J, Horwich A (2006). Molecular chaperones and protein quality control. *Cell*.

[2] Eanes ED, Glenner GG (1968). X-ray diffraction studies on amyloid filaments. *Journal of Histochemistry & Cytochemistry*.

[3] Glenner GG (1980). Amyloid deposits and amyloidosis. The beta-fibrilloses (first of two parts). *The New England Journal of Medicine*.

[4] Westermark P, Benson MD, Buxbaum JN (2005). Amyloid: toward terminology clarification. Report from the Nomenclature Committee of the International Society of Amyloidosis. *Amyloid*.

[5] Puchtler H, Sweat F, Kuhns JG (1964). On the binding of direct cotton dyes by amyloid. *Journal of Histochemistry & Cytochemistry*.

[6] Jarrett JT, Lansbury PT (1993). Seeding “one-dimensional crystallization” of amyloid: a pathogenic mechanism in Alzheimer's disease and scrapie?. *Cell*.

[7] Westermark P (2005). Aspects on human amyloid forms and their fibril polypeptides. *FEBS Journal*.

[8] Arispe N, Rojas E, Pollard HB (1993). Alzheimer disease amyloid *β* protein forms calcium channels in bilayer membranes: blockade by tromethamine and aluminum. *Proceedings of the National Academy of Sciences of the United States of America*.

[9] Caughey B, Lansbury PT (2003). Protofibrils, pores, fibrils, and neurodegeneration: separating the responsible protein aggregates from the innocent bystanders. *Annual Review of Neuroscience*.

[10] Quist A, Doudevski I, Lin H (2005). Amyloid ion channels: a common structural link for protein-misfolding disease. *Proceedings of the National Academy of Sciences of the United States of America*.

[11] Westermark P, Wernstedt C, Wilander E, Sletten K (1986). A novel peptide in the calcitonin gene related peptide family as an amyloid fibril 
protein in the endocrine pancreas. *Biochemical and Biophysical Research Communications*.

[12] Westermark P, Wilander E, Westermark GT, Johnson KH (1987). Islet amyloid polypeptide-like immunoreactivity in the islet B cells of type 2 (non-insulin-dependent) diabetic and non-diabetic individuals. *Diabetologia*.

[13] Westermark P, Grimelius L (1973). The pancreatic islet cells in insular amyloidosis in human diabetic and non-diabetic adults. *Acta Pathologica et Microbiologica Scandinavica A*.

[14] Clark A, Wells CA, Buley ID (1988). Islet amyloid, increased A-cells, reduced B-cells and exocrine fibrosis: quantitative changes in the pancreas in type 2 diabetes. *Diabetes Research*.

[15] Butler AE, Janson J, Bonner-Weir S, Ritzel R, Rizza RA, Butler PC (2003). *β*-cell deficit and increased *β*-cell apoptosis in humans with type 2 diabetes. *Diabetes*.

[16] Mirzabekov TA, Lin MC, Kagan BL (1996). Pore formation by the cytotoxic islet amyloid peptide amylin. *Journal of Biological Chemistry*.

[17] Janson J, Ashley RH, Harrison D, McIntyre S, Butler PC (1999). The mechanism of islet amyloid polypeptide toxicity is membrane disruption by intermediate-sized toxic amyloid particles. *Diabetes*.

[18] Anguiano M, Nowak RJ, Lansbury PT (2002). Protofibrillar islet amyloid polypeptide permeabilizes synthetic vesicles by a pore-like mechanism that may be relevant to type II diabetes. *Biochemistry*.

[19] Rumora L, Hadzija M, Barisic K, Maysinger D, Grubiic TZ (2002). Amylin-induced cytotoxicity is associated with activation of caspase-3 and MAP kinases. *Journal of Biological Chemistry*.

[20] Zhang S, Liu J, Dragunow M, Cooper GJ (2003). Fibrillogenic amylin evokes islet *β*-cell apoptosis through linked activation of a caspase cascade and JNK1. *Journal of Biological Chemistry*.

[21] Lorenzo A, Razzaboni B, Weir GC, Yankner BA (1994). Pancreatic islet cell toxicity of amylin associated with type-2 diabetes mellitus. *Nature*.

[22] Sanke T, Bell GI, Sample C, Rubenstein AH, Steiner DF (1988). An islet amyloid peptide is derived from an 89-amino acid precursor by proteolytic processing. *Journal of Biological Chemistry*.

[23] Badman MK, Shennan KI, Jermany JL, Docherty K, Clark A (1996). Processing of pro-islet amyloid polypeptide (proIAPP) by the prohormone convertase PC2. *FEBS Letters*.

[24] Higham CE, Hull RL, Lawrie L (2000). Processing of synthetic pro-islet amyloid polypeptide (proIAPP) ‘amylin’ by recombinant prohormone convertase enzymes, PC2 and PC3, in vitro. *European Journal of Biochemistry*.

[25] Marzban L, Trigo-Gonzalez G, Zhu X (2004). Role of *β*-cell prohormone convertase (PC)1/3 in processing of pro-islet amyloid polypeptide. *Diabetes*.

[26] Paulsson JF, Westermark GT (2005). Aberrant processing of human proislet amyloid polypeptide results in increased amyloid formation. *Diabetes*.

[27] Marzban L, Rhodes CJ, Steiner DF, Haataja L, Halban PA, Verchere CB (2006). Impaired NH_2_-terminal processing of human pro-islet amyloid polypeptide by the prohormone convertase PC2 leads to amyloid formation and cell death. *Diabetes*.

[28] Paulsson JF, Andersson A, Westermark P, Westermark GT (2006). Intracellular amyloid-like deposits contain unprocessed pro-islet amyloid polypeptide (proIAPP) in beta cells of transgenic mice overexpressing the gene for human IAPP and transplanted human. *Diabetologia*.

[29] Köhler M, Zaitsev SV, Zaitseva II (2003). On-line monitoring of apoptosis in insulin-secreting cells. *Diabetes*.

[30] Schägger H, von Jagow G (1987). Tricine-sodium dodecyl sulfate-polyacrylamide gel electrophoresis for the separation of proteins in the range from 1 to 100 kDa. *Analytical Biochemistry*.

[31] Westermark GT, Gebre-Medhin S, Steiner DF, Westermark P (2000). Islet amyloid development in a mouse strain lacking endogenous islet amyloid polypeptide (IAPP) but expressing human IAPP. *Molecular Medicine*.

[32] Westermark P, Li ZC, Westermark GT, Leckström A, Steiner DF (1996). Effects of beta cell granule components on human islet amyloid polypeptide fibril formation. *FEBS Letters*.

[33] Jaikaran ET, Nilsson MR, Clark A (2004). Pancreatic *β*-cell granule peptides form heteromolecular complexes which inhibit islet amyloid 
polypeptide fibril formation. *Biochemical Journal*.

[34] Kayed R, Head E, Thompson JL (2003). Common structure of soluble amyloid oligomers implies common mechanism of pathogenesis. *Science*.

[35] Glabe CG, Kayed R (2006). Common structure and toxic function of amyloid oligomers implies a common mechanism 
of pathogenesis. *Neurology*.

[36] Butler AE, Janson J, Soeller WC, Butler PC (2003). Increased *β*-cell apoptosis prevents adaptive increase in *β*-cell mass in mouse model of type 2 diabetes. *Diabetes*.

[37] Meier JJ, Kayed R, Lin CY (2006). Inhibition of human IAPP fibril formation does not prevent *β*-cell death: evidence for distinct actions of oligomers and fibrils of human IAPP. *American Journal of Physiology*.

[38] Knight JD, Miranker AD (2004). Phospholipid catalysis of diabetic amyloid assembly. *Journal of Molecular Biology*.

